# Adding nutrients to the biocontrol strain JK-SH007 promotes biofilm formation and improves resistance to stress

**DOI:** 10.1186/s13568-019-0929-8

**Published:** 2020-02-11

**Authors:** Huanhuan Fu, Feifei Chen, Wanhui Liu, Weiliang Kong, Chaoen Wang, Xueqi Fang, Jianren Ye

**Affiliations:** Co-Innovation Center for Sustainable Forestry in Southern China, College of Forestry, Nanjing Forestry University, Nanjing, 210037 China

**Keywords:** *B. pyrrocinia* JK-SH007, Biofilm, Formation condition, RT-qPCR

## Abstract

*Burkholderia pyrrocinia* JK-SH007 is an important biocontrol strain for the prevention and treatment of poplar canker disease. Its powerful biocontrol function is inseparable from its successful colonization of poplar trees. Bacterial biofilms can ensure the long-term colonization of a host. To explore the mechanism of action of biofilms in the biocontrol process, we manipulated various exogenous factors to explore the morphology of the JK-SH007 biofilm in vitro. The addition of glycerol and MgSO_4_ to TSB medium stimulated biofilm production, increased the resistance of JK-SH007 to disease, enhanced the survival of JK-SH007 in nutrient-poor environments and maintained the antagonistic ability of JK-SH007 against the poplar canker pathogen. Therefore, we constructed and optimized a biofilm-forming system to produce a large number of stable JK-SH007 biofilms. The optimized system showed that the optimal incubation time for JK-SH007 biofilm formation was 14 h, the optimal temperature of the static culture was 25 °C, and the optimal pH was 5. The optimal medium for biofilm formation was TSB medium, 1% glycerol and 50 mM MgSO_4_. RT-qPCR experiments showed that an increase in the expression of the *suhB* gene promoted JK-SH007 biofilm formation, while an increase in the expression level of the *ropN* gene inhibited JK-SH007 biofilm formation. The possible mechanism by which JK-SH007 was inhibited by biofilm formation under natural culture was revealed. These results indicate the importance of adding nutrients to JK-SH007 biocides produced on a commercial scale. This is the first report of JK-SH007 producing a long-lasting biofilm that guarantees antagonism.

## Introduction

Bacterial biofilms are organized and distinct three-dimensional structures of life groups (Mah et al. 2003). The resistance of bacteria in this state is much stronger than that in the planktonic state, and bacteria in this state have the advantages of sensing the surrounding environment and rapidly ingesting nutrients (Song et al. 2018). Statistically, most bacteria in nature exist in biofilms (Whitchurch et al. 2002). There is evidence that the formation of a biofilm is an important step in the sustainable development and spread of bacteria (Guilhen 2017), including the exchange of intermediate genes and their synergy (Davey and O’Toole 2000). In 1995, Costerton first proposed the concept of a biofilm (Costerton et al. 1995). Later, in research of the interaction between plants and bacteria, researchers found that biofilms are particularly important for the colonization of bacteria, and biofilms have since become a research hotspot in related fields (Yang et al. 2017).

In the process of colonizing host plants and bacteria, bacteria can form biofilms on their surfaces. These biofilms are described as microcolonies, aggregates, cell clusters, etc. (Chang and Halverson 2003). Biocontrol bacteria have a significant promoting effect on colonization in the environment surrounding plants. For example, the formation of biofilms greatly promotes the colonization of *Bacillus subtilis* in Arabidopsis roots and protects Arabidopsis roots from pathogens, such as *Aureobasidium pullulans*. The formation of biofilms in citrus improves the control of citrus soft diseases (Klein and Kupper 2018). Other studies have shown that some biocontrol bacteria can secrete important components of synthetic biofilms in plants, e.g., extracellular polysaccharide (Eps), which facilitates the colonization on the root hairs and roots of legumes (Fall and Vivanco 2004; Chen et al. 2009; Fujishige et al. 2010).

Currently, in the research of gram-negative biocontrol bacteria that are beneficial to humans, the biofilm morphology of many strains under normal artificial culture has not been successfully observed, and related research is rarely reported. *Burkholderia pyrrocinia* JK-SH007, studied in this paper, is a highly efficient gram-negative biocontrol agent involved in the interaction between plants and bacteria of poplar and belongs to the *Burkholderia cepacia* complex (Bcc) Genotype IX (Chen et al. 2018). *Burkholderia cepacia* epidemic strain marker (BCESM) and *cblA* virulence gene-specific PCRs in addition to the commonly used safety detection techniques for Bcc such as the *Allium cepa*, *Nicotiana* sp. and *Medicago* sp. models were used to detect strain toxicity and indicated that JK-SH007 is a potentially safe biocontrol strain (Jia-Hong et al. 2010). The results of previous studies showed that the strain had a strong antagonistic effect on three pathogens of poplar canker, *Phomopsis macrospora*, *Cytospora chrysospermus* and *Fusicoccum aesculi*, and can also promote plant growth by secreting growth hormones, such as IAA, polygalacturonase and ferritin (Ren et al. 2011; Yang et al. 2017). However, little is known about the morphology of the biofilm of this strain. In this study, JK-SH007 was successfully cultured by changing the conditions of exogenous stress. The difference in stress resistance and related gene expression between planktonic bacteria and biofilm bacteria was studied. JK-SH007 was more capable of dormancy or long-term survival in the biofilm state than in the planktonic state.

## Materials and methods

### Experimental materials

*Burkholderia pyrrocinia* JK-SH007 (strain number: CCTCC M209028) was acquired from Jiangsu Provincial Key Laboratory of Pest Invasion Prevention and Control. Highly aggressive isolates of *Phomopsis macrospora* (strain number: CCTCC AF 2015001) were isolated from naturally infected poplar stem and maintained on potato dextrose agar (PDA) slants at 4 °C in laboratory (Wang and Wu 2008).

LB medium (10 g/L tryptone, 5 g/L yeast extract, and 10 g/L NaCl), TSB medium (15 g/L tryptone, 5 g/L soya peptone, and 5 g/L NaCl), and the crystal violet solution were prepared manually, and ethanol, glycerol, MgSO_4_·7H_2_O, K_2_HPO_4_, FeCl_3_·6H_2_O, and CaCl_2_ were all commercially available.

### Preparation of the *B. pyrrocinia* JK-SH007 suspension

The JK-SH007 strain was grown on LB solid plate medium to form single colonies. Single colonies were picked, transferred to LB liquid medium and cultured at 180 r/min and 28 °C for 24 h to obtain a bacterial suspension.

### Study of the conditions for JK-SH007 biofilm formation

Fifteen different test media were prepared according to Table 1. The bacterial suspension was inoculated at 1%, and the amount of liquid was 20 mL (50 mL triangular bottle). The cells were cultured at 28 °C and 180 r/min for a specified period of time and then moved to a 12-well cell culture plate for static culture.

**Table 1:** Formulas of the 15 tested media

Serial number	Tryptone (g)	Yeast extract (g)	Soya peptone (g)	Glucose (g)	NaCl (g)	Glycerol (mL)	MgSO_4_·7H_2_O (g)	K_2_HPO_4_ (g)	FeCl_3_·6H_2_O (g)	CaCl_2_ (g)
1	10	5			10					
2	10	5			10	10	6.1617			
3	10	5			10	10			6.7572	
4	10	5			10	10		5.7055		
5	10	5			10	10				2.7747
6	10	5			10	10				
7	15		5		5					
8	15		5	10	5					
9	15		5		5	10				
10	15		5		5	10	6.1617			
11	15		5		5	10		5.7055		
12	15		5		5	10			6.7572	
13	15		5		5	10				2.77475
14	10	5		10	10					
15	10	5		10	10	10				

### Morphological observation and related phenotypic determination of the biofilm state of JK-SH007

TSB medium was selected as a negative control group (Control), which did not form a biofilm, and Mg^2+^ and glycerol were added to TSB medium to form a biofilm-forming treatment group (TMG). The morphological differences in JK-SH007 in the planktonic and biofilm states were observed.

The cells were inoculated at a 1% bacterial suspension into the two groups and cultured at 180 r/min in a bacterial shaker at 28 °C. The OD_600_ value at 2, 4, 6, 8, 10, 12, 14, and 24 h was measured using an ultraviolet (UV) spectrophotometer, and a growth curve table was drawn. A small amount of the bacterial suspension was taken for the kinetic determination of JK-SH007 in the swimming medium, and the swimming ability of the strain under the planktonic and biofilm states was observed (Zhang 2016). Another microbacterial suspension was removed for the determination of the antagonistic ability of the bacteria against pathogenic bacteria.

After cultures were allowed to stand for 6 days, the two groups of bacterial suspensions were removed, and the OD_600_ value was measured to calculate the total number of cells. The gradient concentration was diluted, and 100 µL of the diluted sample was plated on LB solid medium (Fan et al. 2018); the number of active bacteria was counted 24 h later.

After cultures were allowed to stand for 6 days, the biofilm is extracted after the culture was allowed to stand for 6 days. The biofilm is weighed after drying, followed by dissolved in distilled water. A gradient dilution is performed to obtain the test sample. Anthrone-sulfuric acid method (Xi-Feng et al. 2017) was used to draw a sugar standard curve to determine the extracellular polysaccharide content.

1 $$ {\text{N}} = 1. 6 30 3 {\text{X }} + 0.0 5 1 4 {\text{ R}}^{ 2} = 0. 9 9 4 8 $$

2 $$ {\text{V}} = \left( {{\text{d}}_{ \hbox{max} } + {\text{d}}_{ \hbox{min} } } \right)/ 4 { } \times {\text{t}} $$

3 $$ {\text{Y = 51}} . 3 3 8 {\text{a}} - 9. 3 0 2 4 {\text{ R}}^{ 2} { = 0} . 9 9 7 7 $$

4 $$ {\text{C}} = {\text{a}}/{\text{b }} \times 100\% $$

(N represents the total number of bacteria; X represents the absorbance of the bacterial suspension at 600 nm in a UV spectrophotometer; V represents the swimming rate; d_max_ indicates the maximum diameter of the bacteria swimming in the plate medium; and d_min_ indicates the minimum diameter of the bacteria swimming in the plate medium; Y is glucose content;a represents the absorbance of the bacterial suspension at 625 nm in a UV spectrophotometer; C is extracellular polysaccharide mass fraction; b is sample mass).

### Semiquantitative evaluation of *B. pyrrocinia* JK-SH007 biofilm

The bacterial suspension was inoculated at 1% into TMG medium for exponential culture. After the logarithmic phase was reached, 3 ml of the exponential suspension was transferred to a 12-well cell culture plate, and the number of days the culture was allowed to stand was 2, 4, 6, 8, 10, 12, 14, and 16 days. Biofilm formation was measured by crystal violet staining.

### Effect of standing time on the amount of *B. pyrrocinia* JK-SH007 biofilm formed

The bacterial suspension was inoculated at 1% in TMG medium for exponential culture. After the logarithmic phase was reached, 3 mL of the exponential suspension was transferred to a 12-well cell culture plate, and the number of days the culture was allowed to stand was 2, 4, 6, 8, 10, 12, 14, and 16 days. Biofilm formation was measured by crystal violet staining.

### Effect of temperature on the formation of the *B. pyrrocinia* JK-SH007 biofilm

The bacterial suspension was inoculated in TMG medium at 1%, and after the logarithmic phase, 3 mL of the exponential suspension was transferred to a 12-well cell culture plate. The culture was allowed to stand at a temperature of 15 °C. The cells were cultured for 4–5 days at 20 °C, 25 °C, 30 °C, and 35 °C. After a stable biofilm was formed, the amount of biofilm formed was determined by crystal violet staining.

### Effect of pH on the formation of the *B. pyrrocinia* JK-SH007 biofilm

The bacterial suspension was inoculated in at 1% in TMG medium for exponential culture. The pH of the medium was adjusted to 3, 4, 5, 6, 7, 8, 9, and 10 using HCl/NaOH. The exponentially growing bacterial suspension was transferred to a 12-well cell culture plate at 3 mL per well, and after a stable biofilm was formed, the amount of biofilm formed was measured by crystal violet staining.

### Orthogonal experimental design

Exploratory experiments indicated that three factors play an important role in biofilm formation: Glycerol concentration, culture time, and Mg^2+^ concentration. Therefore, we conducted a three-factor and three-level orthogonal experiment at Table 2 (Wang et al. 2014), taking JK-SH007 biofilm formation as the main investigation. Indicators were used to determine the best ratio system.

**Table 2:** Training conditions of the orthogonal factor level table

Level/factor	A (Glycerol)/(vol/vol)	B (Training time)/(h)	C (Mg^2+^)/(mM)
1	0.10%	8	0.5
2	1%	14	5
3	10%	20	50

### Primer design

Biofilm-related gene (*suhB* and *ropN*)-specific primers were designed using primer5.0 according to the sequencing and genetic annotation of *Burkholderia pyrrocinia* JK-SH007 (Wang et al. 2018), as shown in Table 3. The primers were synthesized by GenScript.

**Table 3:** Primer sequences used for gene amplification

Primer	Sequence (5′ → 3′)	Bases (bp)
*ropN*-F	ATGAAGCACACGCTCTCCCTCG	22
*ropN*-R	TGCCGGCTGTACGCGATGA	19
qPCR-*suhB*-F	CGTCGGCAGCAACACGTAT	19
qPCR-*suhB*-R	GCGGATCGAGGTTGTAGATC	20
qPCR-*ropN*-F	GAGATCGACTGCGACGAACTG	21
qPCR-*ropN*-R	CAGCTCCAGATGCTGCGAC	19
*pyrG2*-F	AGTCACCCTCCTCAAACTCG	20
*pyrG2*-R	TCGTGAAGTTGTTGGCCTTG	20

### *Burkholderia pyrrocinia* JK-SH007 total DNA extraction, total RNA extraction and cDNA synthesis

Total DNA extraction: The bacterial suspension was collected, and the JK-SH007 genomic DNA was extracted by the modified Cetyl Trimethyl Ammonium Bromide (CTAB) method and detected by UV spectrophotometer and agarose gel electrophoresis. After the required experimental concentration was obtained, the extracted DNA was stored at − 80 °C.

Synthesis of total RNA and cDNA: According to the Trizol reagent specification (TaKaRa), the total RNA of the JK-SH007 strain in the two steps of the 1.3 step was cultured for 14 h and allowed to stand for 6 days to form a biofilm. UV spectrophotometer and agarose gel electrophoresis detection were performed, and after the required experimental concentration was reached, the samples were stored at − 80 °C. cDNA was generated with the HiScript II^®^ Reverse Transcriptase R Kit (Vazyme-innovation in enzyme technology), and the corresponding cDNA was reverse transcribed using a random primer and stored in a − 20 °C refrigerator for use.

### *Burkholderia pyrrocinia* JK-SH007 biofilm-related gene cloning and differential expression analysis

The extracted DNA was used as a template to amplify the *suhB* and *ropN* genes using a common Taq enzyme. The final volume of the PCR was 10 µL and included the following: 5 µL common ExTap enzyme, 0.5 µL DNA template, 0.5 µL forward primer, 0.5 µL reverse primer, and 3.5 µL ddH_2_O. The PCR system was as follows: 95 °C for 10 min; 30 cycles of 95 °C for 30 s, 61 °C for 30 s, and 72 °C for 2 min; and 72 °C for 10 min. After the reaction, the PCR product was detected by 1% agarose gel electrophoresis. The obtained target fragment was ligated into the pMDTM19-T vector and transferred into *E. coli* JM109 to obtain a positive clone sequence. The gene of the positive close was sequenced at Nanjing Huada Gene, and the sequencing results confirmed the gene (Meng et al. 2017).

The cDNA from the biofilm treatment was used as the TMG template, and the cDNA from the biofilm treatment was not used as the Control template; the ChamQ™ SYBR^®^ qPCR Master Mix Kit (Vazyme-innovation in enzyme technology) was used according to the manufacturer’s manual. Quantitative analysis was performed with *pyrG2* as the internal reference gene primer, and the qPCR-specific primers in Table 3 were the *suhB* and *ropN* gene primers. Quantitative PCRs were performed on an ABI 7500 real-time PCR instrument (Zhou et al. 2018). The reaction system was 20 µL, including 10 µL of ChamQ SYBR qPCR Master Mix (without ROX), 1.5 µL of each of the different primers, 1 µL of cDNA template, and 6 µL of ddH_2_O. The amplification procedure was predenaturation at 95 °C for 10 s, and 40 cycles of denaturation at 95 °C for 10 s and annealing at 60 °C for 30 s. The relative expression levels of the *suhB* and *ropN* genes were calculated by the 2^−ΔΔCT^ method (Pourhajibagher et al. 2018).

### Data analysis

The data were analyzed using Excel 2010, SPSS 20.5, OriginPro8.6 data analysis and statistical software.

## Results

### Evaluation of *B. pyrrocinia* JK-SH007 biofilm formation

#### Determination of the test medium

**Fig. 1:**
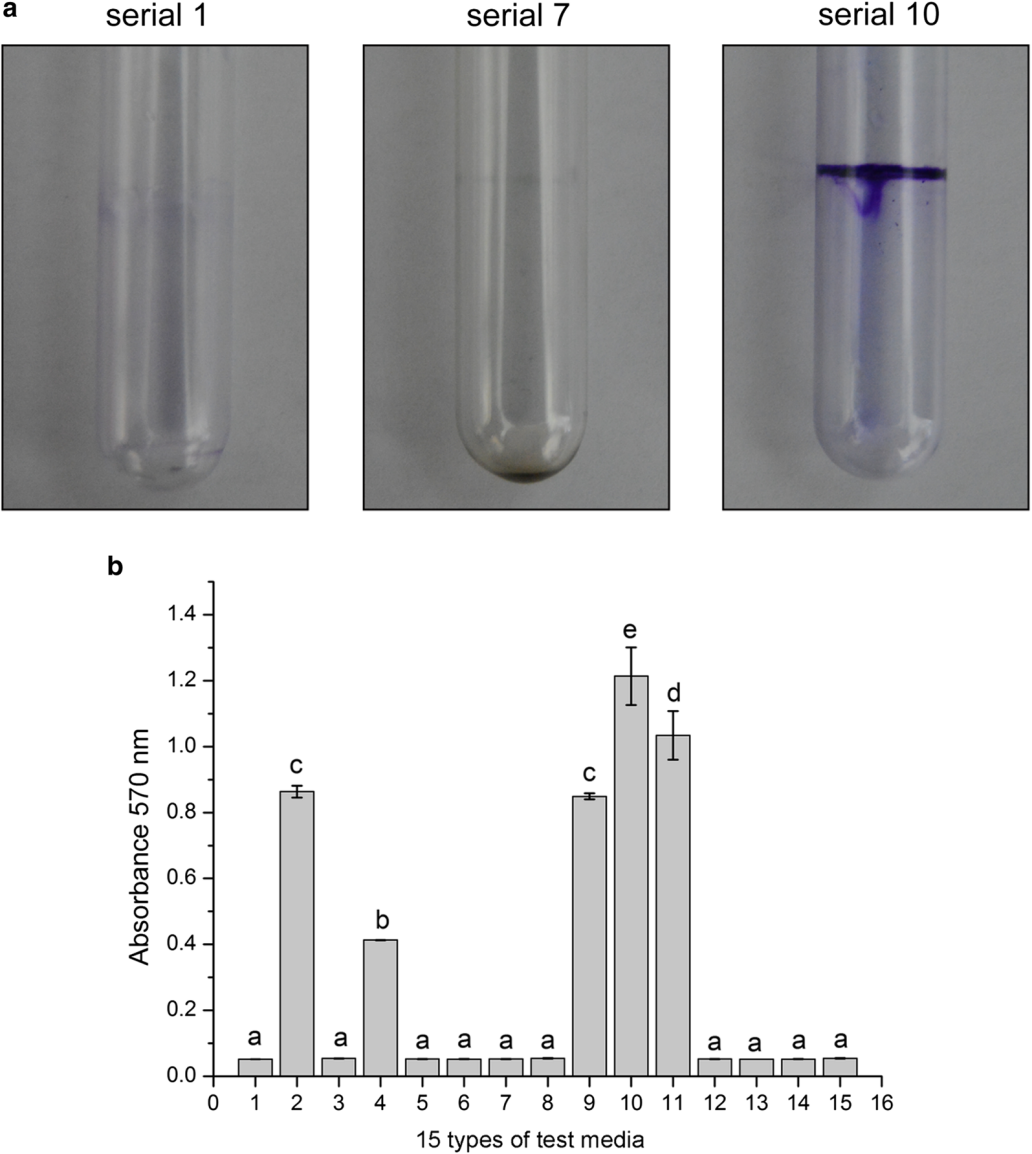
Effects of different test media on JK-SH007 biofilm formation. **a** Images of crystal violet-stained biofilms formed by serial 1, serial 7 and serial 10. Cells were grown in test media for 6 days at 20 °C under static conditions before crystal violet staining. **b** Quantitative comparison of biofilm formation in serial 1, serial 7 and serial 10. Each experiment was performed at least three times in triplicate. Error bars represent the standard error of the mean. Different lowercase letters represent significant differences (P < 0.05)

Fifteen different test media had a substantial influence on JK-SH007 biofilm formation. JK-SH007 could not form a biofilm in static culture in No. 1 medium (LB) and No. 7 medium (TSB), and test media No. 2, No. 4, No. 9, No. 10 and No. 11 resulted in the formation of biofilms that were visible to the naked eye. Among them, the No. 10 test medium formed the largest amount of biofilm (Fig. 1), which was the TSB medium + glycerol + MgSO_4_ system. The biofilm-forming ability of the test medium system based on LB medium was generally weak, and under the same exogenous substance stress, the amount of biofilm formation was lower in LB than in TSB medium.

### Effect of metal ions on the formation of *B. pyrrocinia* JK-SH007 biofilms

**Fig. 2:**
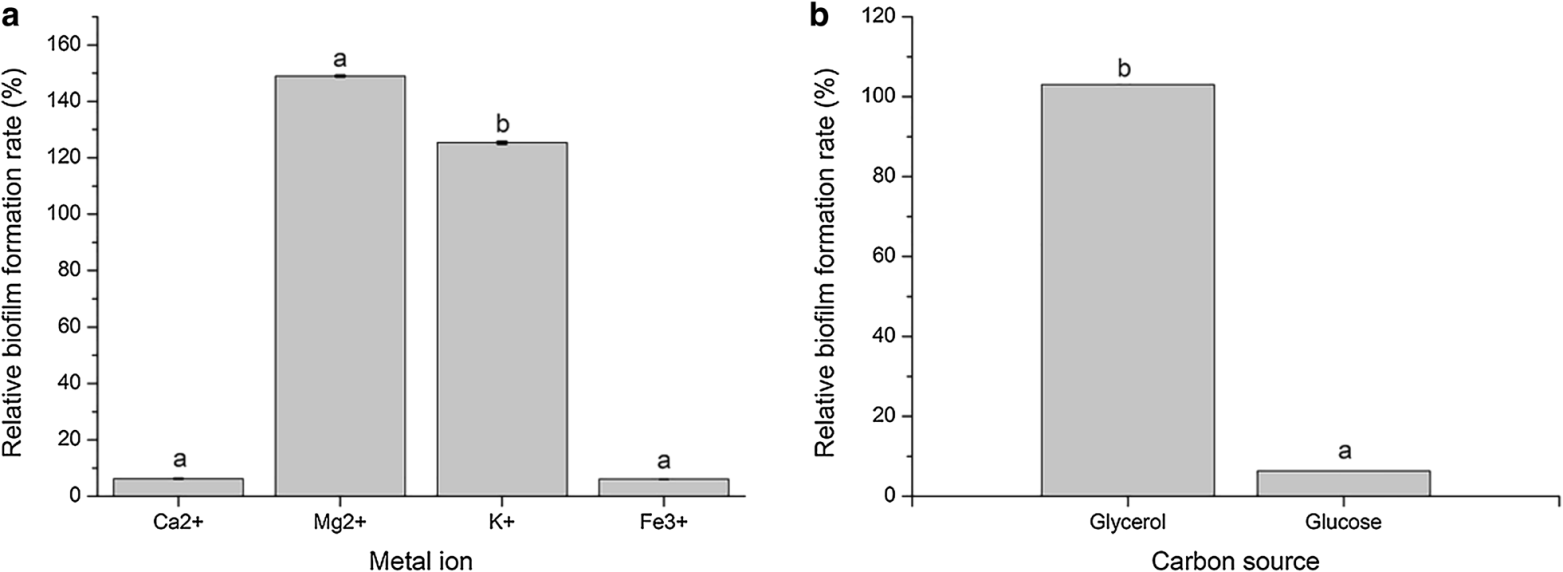
**a** The effects of different metal ions on the ability of JK-SH007 to form biofilms. **b** The effects of two carbon source materials on the ability of JK-SH007 to form biofilms. Different lowercase letters represent significant differences (P < 0.05)

Ca^2+^ and Fe^3+^ did not significantly promote JK-SH007 biofilm formation. In contrast, Mg^2+^ and K^+^ significantly promoted biofilm formation, and Mg^2+^ had the best effect on biofilm formation (Fig. 2a).

### Effect of two carbon source materials on the formation of *B. pyrrocinia* JK-SH007 biofilms

The addition of glycerol to the culture medium resulted in the production of a mature and visible biofilm. The relative biofilm formation reached 100%, and the addition of glucose to the medium did not significantly promote the formation of the biofilm of the bacteria (Fig. 2b). Glycerol is a major factor in the formation of JK-SH007 biofilms.

### Observation of *B. pyrrocinia* JK-SH007 biofilm morphology and the determination of the viable bacteria rate

**Fig. 3:**
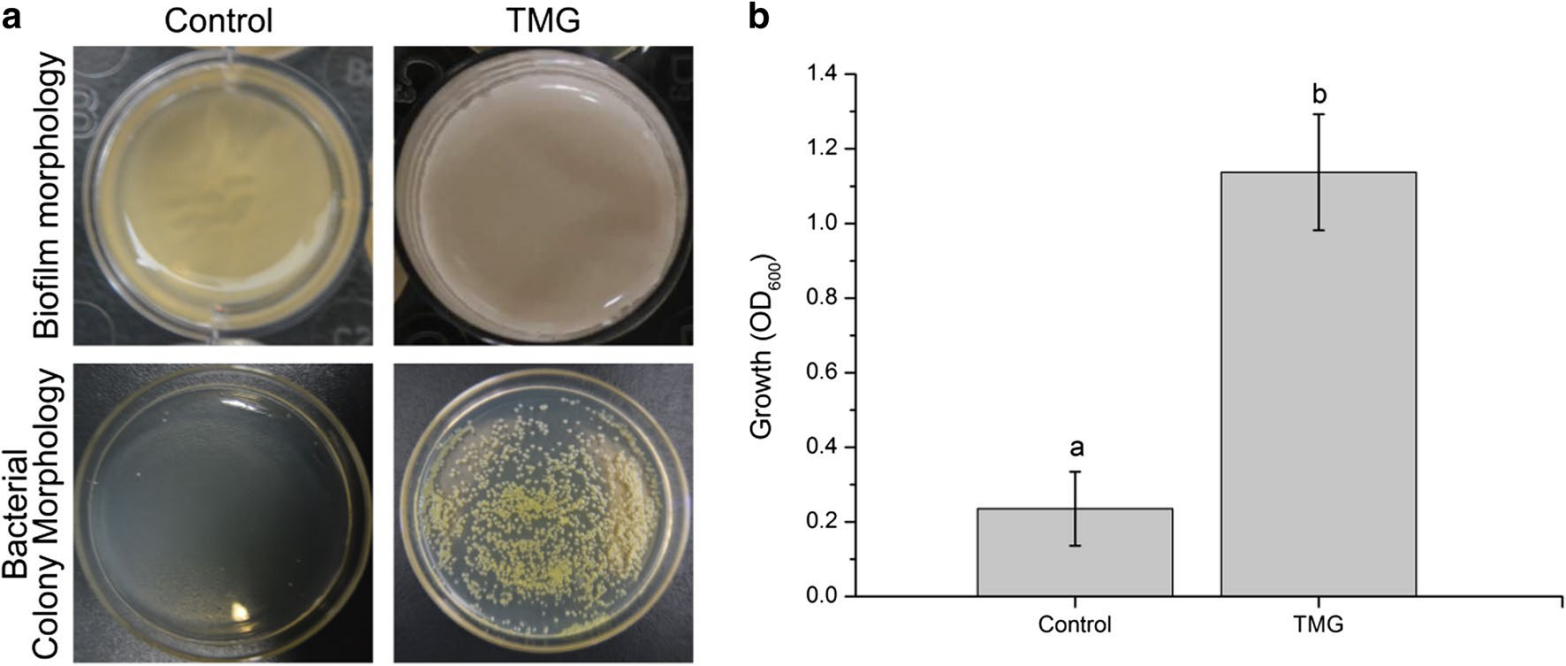
**a** Phenotype and activity ability of planktonic bacteria and biofilm bacteria. **b** OD600 value of JK-SH007 in suspension after 6 days of standing. Each experiment was performed at least three times in triplicate. Error bars represent the standard error of the mean. Different lowercase letters represent significant differences (P < 0.05)

As shown in Fig. 3a, the biofilm of the JK-SH007 TMG was white overall; the surface of the membrane was smooth and closely adhered to the interface of the bacterial liquid and air and had a typical three-dimensional biofilm structure. After the Control was allowed to stand for 6 days, the bacterial liquid became clear, and a large amount of dead bacteria sank to the bottom of the culture. The absorbance measurement results of the bacterial suspensions after treatment for 6 days in both groups are shown in Fig. 3b. The OD_600_ value of the TMG was as high as 1.137, while the Control group suspension had an OD_600_ value of only 0.235. The number of active bacteria in the TMG was approximately 4.1 × 10^10^ CFU/mL, and the total number of cells was approximately 2.1417 × 10^12^ CFU/mL. The number of active bacteria in the Control group was approximately 9 × 10^5^ CFU/mL, and the total number of cells was approximately 4.3 × 10^8^ CFU/mL.

### Determination of growth curves, EPS, antagonistic abilities and motility differences

**Fig. 4:**
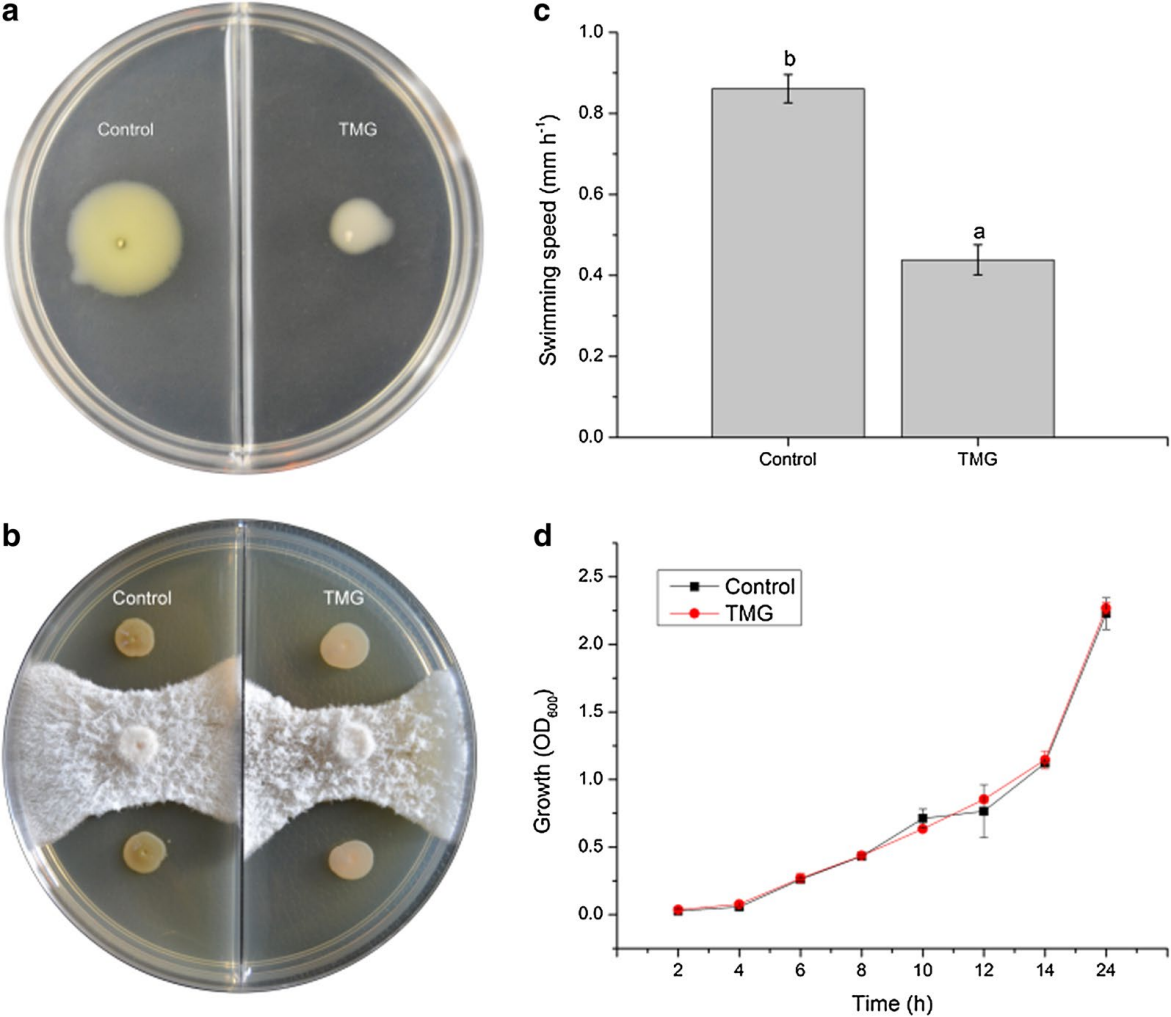
**a** Observation of the movement of JK-SH007 in two swimming media. **b** Comparison of the antagonistic capacity of JK-SH007 in two states. **c** Differences in growth curves between the biofilm state and planktonic JK-SH007. **d** Differences in the motility of JK-SH007 biofilm bacteria and planktonic bacteria. Each experiment was performed at least three times in triplicate. Error bars represent the standard error of the mean. Different lowercase letters represent significant differences (P < 0.05)

The addition of appropriate concentrations of Mg^2+^ and glycerol greatly reduced the swimming capacity of JK-SH007 (Fig. 4a), The added nutrients penetrated the biofilm but did not alter the antagonistic ability of JK-SH007. In contrast, the formation of a biofilm contributes to the long-term survival of active bacteria, resulting in a weak increase in antagonistic capacity (Fig. 4b). which facilitated the aggregation of planktonic bacteria and the formation of biofilms. The activity rate of JK-SH007 was 0.86 mm/h in the Control and 0.44 mm/h in the TMG group by swimming motility measurements (Fig. 4c). To explore the effect of JK-SH007 on the growth rate of biofilm formation, we compared the growth curve of this strain in the Control and TMG. The results are shown in Fig. 4d. The two growth curves are basically the same, indicating that the addition of appropriate concentrations of Mg^2+^ and glycerol did not cause a delay or promotion of the growth of the strain. As for the determination of exopolysaccharide, OD_625_ was 0.4126, and the mass fraction c of exopolysaccharide was 23.8%.

### Effect of rest time on the amount of *B. pyrrocinia* JK-SH007 biofilm formed

**Fig. 5:**
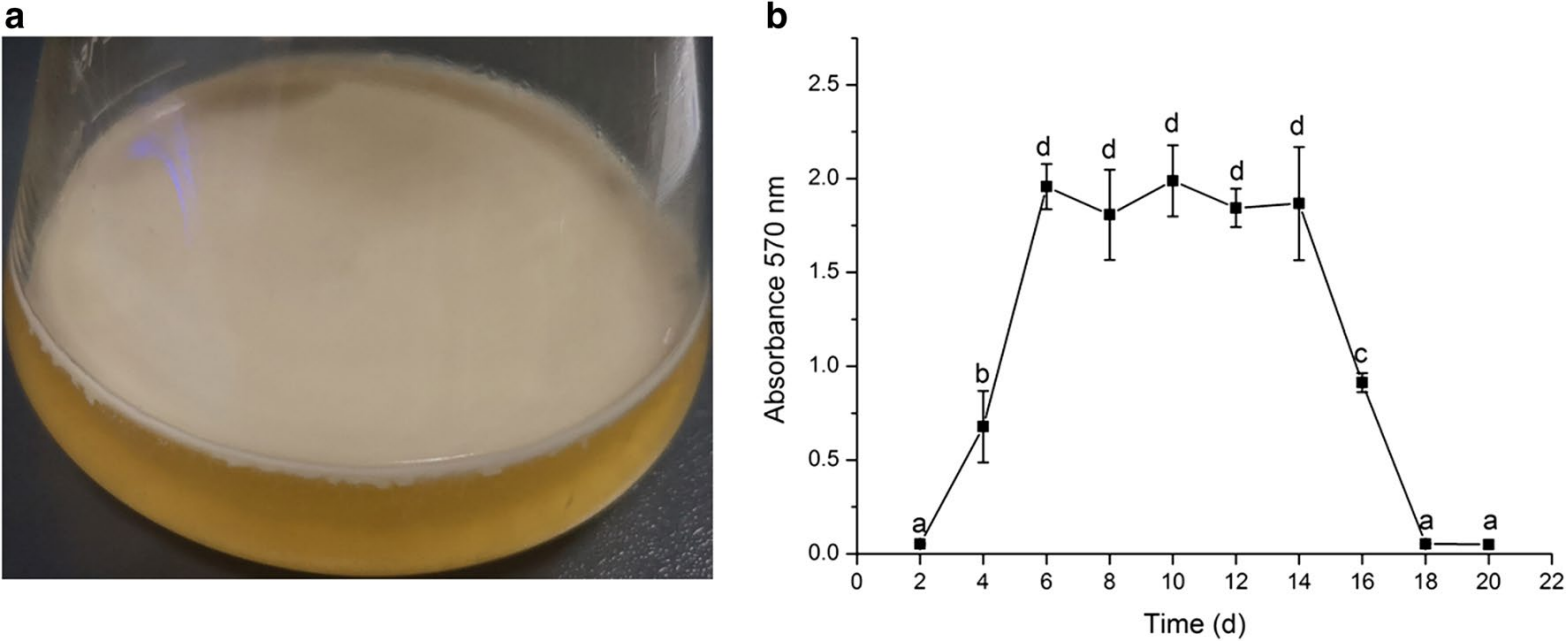
**a** JK-SH007 biofilm in liquid medium. **b** Antagonistic activities of JK-SH007 toward pathogens of poplar canker disease on plates. Differences in biofilm volume at different time periods in the life cycle of the JK-SH007 biofilm. Each experiment was performed at least three times in triplicate. Error bars represent the standard error of the mean. Different lowercase letters represent significant differences (P < 0.05)

The experimental results show that JK-SH007 has a typical life cycle of a bacterial biofilm. Two days before standing, the OD_570_ value was close to 0, indicating no obvious biofilm formation, in the early stage of biofilm formation; within 2–6 days, the OD_570_ value increased rapidly, indicating that the bacteria rapidly formed a large number of biofilm structures. At the midterm of biofilm formation, a large number of stable biofilms were also observed to adhere to the gas–liquid junction in liquid medium (Fig. 5a). Then, the OD_570_ value was basically stabilized at 6–14 days, and the biofilm was in a mature and stable stage. The OD_570_ value decreased rapidly in the last 14–18 days, indicating that the biofilm began to degrade and eventually disappeared; the bacteria subsequently died (Fig. 5b).

### Effect of temperature conditions on the formation of the *B. pyrrocinia* JK-SH007 biofilm

**Fig. 6:**
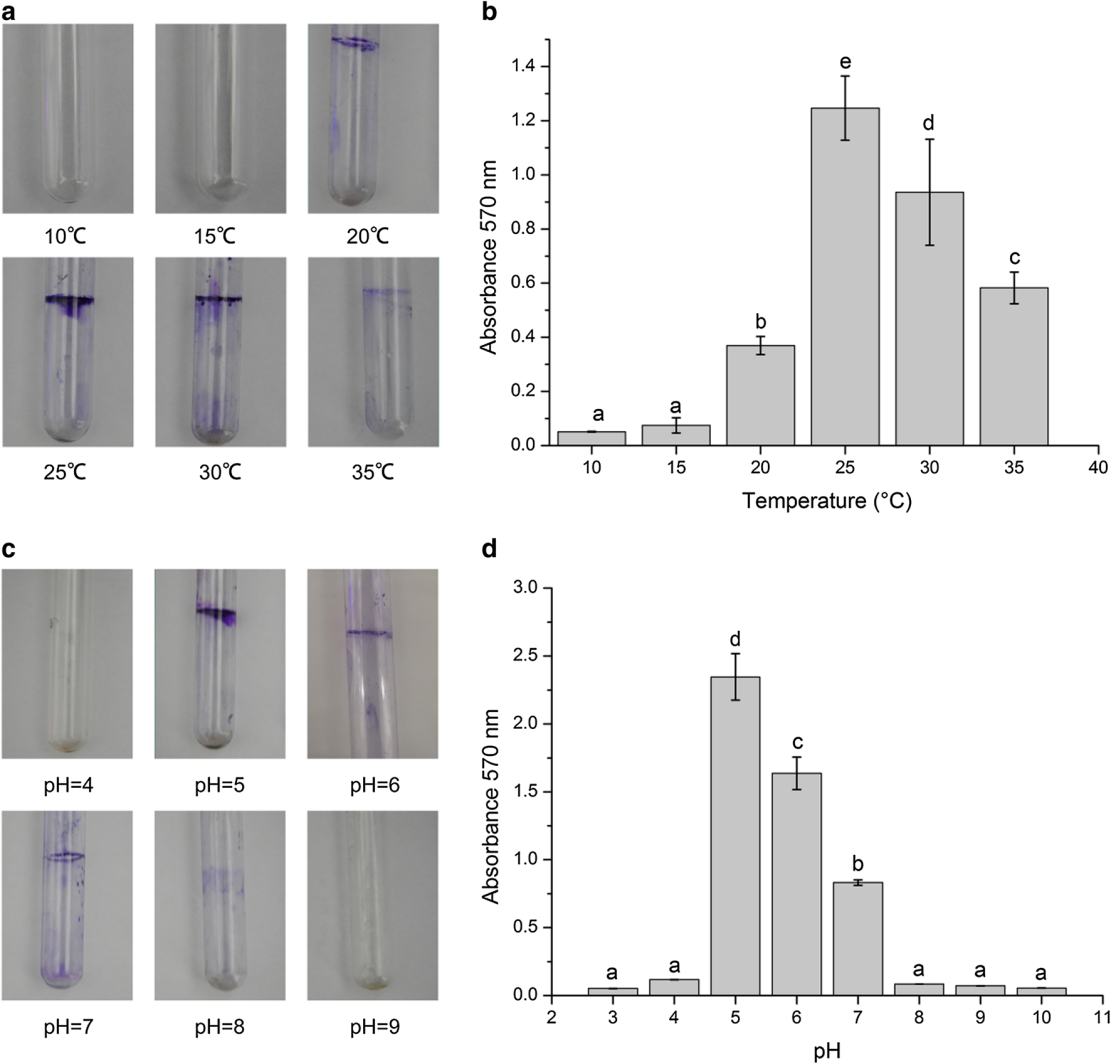
Single factor experiment of JK-SH007 biofilm formation. **a** Image of crystal violet-stained biofilm formed by JK-SH007 at different temperatures. **b** Biofilm formation ability of JK-SH007 under different temperature conditions. **c** An image of a crystal violet-stained JK-SH007 biofilm formed at different pH values. **d** Biofilm formation ability of JK-SH007 under different pH conditions. Each experiment was performed at least three times in triplicate. Error bars represent the standard error of the mean. Different lowercase letters represent significant differences (P < 0.05)

Different temperatures greatly influenced the JK-SH007 biofilm formation. The effect is shown in Fig. 6a, b. Under static culture at 25 °C, the measured OD_570_ value reached a maximum value, and the ability to statically culture the formed biofilm under this temperature condition was optimal. When the temperature was 15 °C and 35 °C, the measured OD_570_ values were the smallest, and the biofilm-forming ability was the weakest; this indicates that a temperature that is too low or high has an inhibitory effect on the formation of the JK-SH007 biofilm.

### Effect of pH conditions on the formation of the *B. pyrrocinia* JK-SH007 biofilm

Different pH values greatly influenced biofilm formation, and the results are shown in Fig. 6c, d. When the pH was 3 or 10, the OD_570_ value was almost zero, and in the 12-well cell plate, the JK-SH007 strain did not grow because the optimum pH range for the growth of the strain was 3.8–9. When the pH was 5, the amount of biofilm formation reached a maximum. After that, as the pH value increased, the amount of biofilm formation slowly decreased.

### Orthogonal experiment of exogenous conditions

Three-factor and three-level orthogonal experiments were performed on the basis of the initial screening of the 15 test media. Taking the amount of biofilm formed at OD_570_ as an indicator, a variance analysis was used to analyze the variance. From Table 4, the main order of factors affecting the formation of *B. pyrrocinia* JK-SH007 biofilm was A > C>B. Its optimal biofilm formation was A_2_B_2_C_3_, indicating that the optimal culture time is 14 h (absorbance 600 nm ≈ 1.1), the amount of glycerol to add is 1% (vol/vol), and the concentration of Mg^2+^ is 50 mM.

**Table 4:** Analysis of orthogonal experimental results of exogenous conditions

Experiment number	Factor level			
	Glycerol (vol/vol)	Training time (h)	C (Mg^2+^)(mM)	OD_570_
1	1	1	1	0.0541
2	1	2	2	0.0973
3	1	3	3	0.1013
4	2	1	2	1.0413
5	2	2	3	1.2146
6	2	3	1	0.5421
7	3	1	3	0.8642
8	3	2	1	0.9421
9	3	3	2	1.1241
K1	0.0842	0.6532	0.5128	
K2	0.9327	0.7513	0.7542	
K3	0.9768	0.5892	0.7267	
Range	0.8926	0.1622	0.2415	
Factor	A > C>B			
Optimal combination	A_2_B_2_C_3_			

### *Burkholderia pyrrocinia* JK-SH007 biofilm-related gene cloning and differential expression analysis

**Fig. 7:**
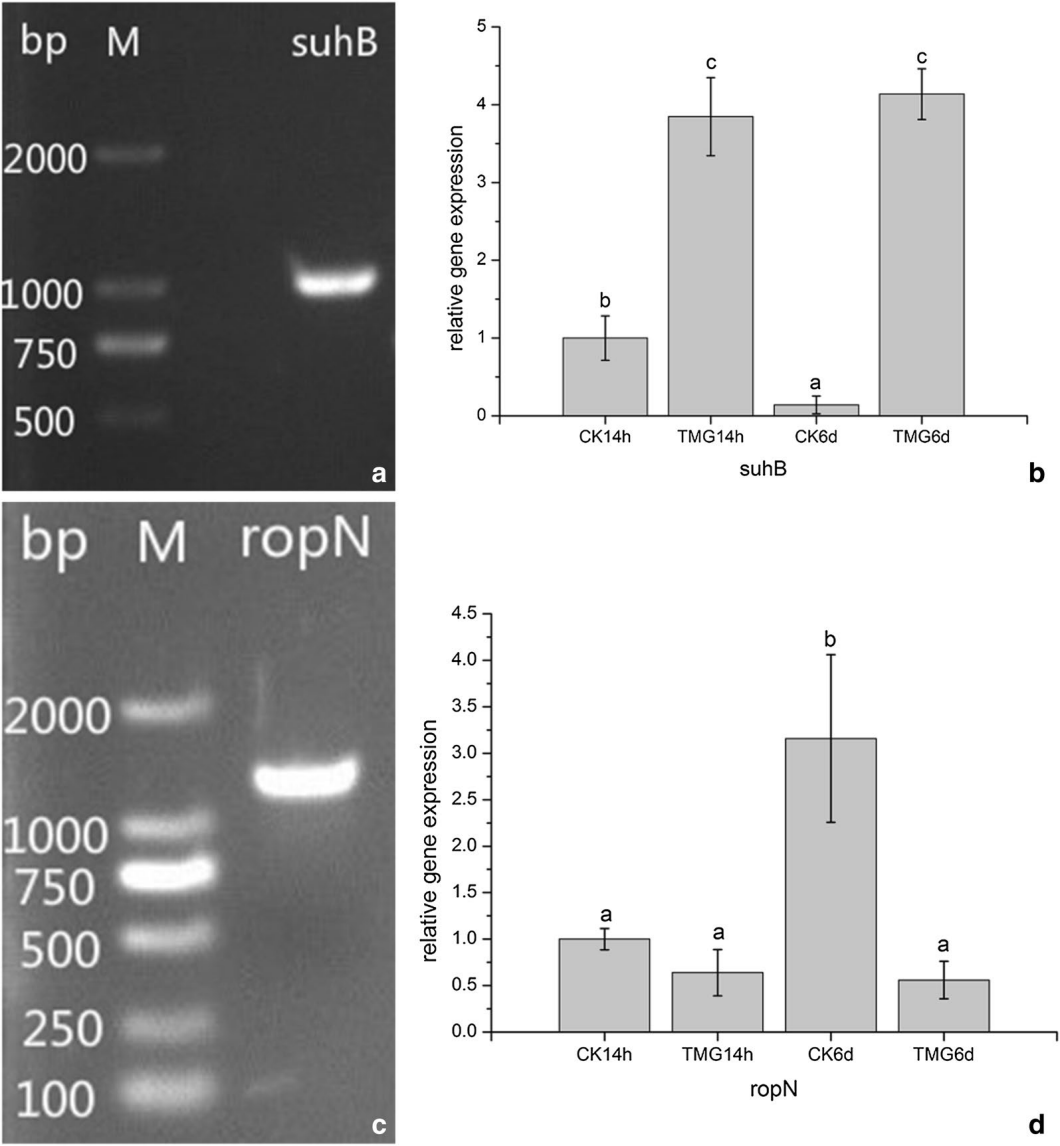
Cloning and differential expression analysis of the *suhB* gene and *ropN* gene. **a** The *suhB* gene of JK-SH007 (M: DL2000 marker). **b** The expression of the *suhB* gene in different treatment time periods. **c** The *ropN* gene of JK-SH007 (M: DL2000 marker). **d** The expression of the ropN gene in different treatment time periods. Each experiment was performed at least three times in triplicate. Error bars represent the standard error of the mean. Different lowercase letters represent significant differences (P < 0.05)

The modified CTAB method was used to extract the DNA of the genome from a JK-SH007 culture after 24 h of shaking. After electrophoresis, the specific primers *suhB*-F/R and *ropN*-F/R, listed in Table 3, were used for PCR amplification, and the sizes of the fragments were obtained. The target fragments were 1100 bp and approximately 1400 bp (Fig. 7a, c).

The analysis of the expression level of biofilm-related genes in the JK-SH007 culture process revealed that the relative expression level of the *suhB* gene was steadily upregulated in the TMG compared with the Control group (CK14h) in the process of biofilm formation. The relative expression was 3.847 at 14 h and 4.137 at 6 days. The relative expression of the *ropN* gene in the TMG group was steadily downregulated and was 0.640 at 14 h and 0.560 at 6 days. The data were significantly different by SPSS 20.0 software analysis (P < 0.05). This indicates that these two genes are closely related to JK-SH007 biofilm formation (Fig. 7b, d).

## Discussion

The biofilm morphology of most gram-negative bacteria studied in the field of forestry is difficult to observe under artificial culture conditions. In this experiment, the biofilm morphology of JK-SH007 was observed for the first time using the exogenous stress method. The new findings fill some gaps in the research on whether gram-negative biocontrol bacteria utilize biofilms for enhanced colonization of their host and have great significance for bacterial-related biofilm research. The formation of the JK-SH007 biofilm mainly occurs in five stages: Cell surface adhesion; monolayer cell population formation; multilayer cell community formation; the formation of a large amount of extracellular material to form a mature biofilm structure; and the dissipation of the mature biofilm (Chen 2012). Bacteria survive for a long time in a biofilm state and maintain certain activities. This is similar to the fact that some of the strains of Onion Burke discovered by Peeters et al. are more likely to survive in a biofilm state than in a planktonic state (Lechugaballesteros et al. 2009). If JK-SH007 can colonize a poplar in a biofilm state, it can maintain stable activity in the poplar for a long time and improve the biocontrol ability against poplar canker. As a biocontrol bacterium, its greatest significance is the ability of antagonize pathogens. In the antagonism experiment, its core traits are ensured not to inhibited while changing some of its traits. Through the antagonistic experiments had proven that the formation of biofilms does not affect its antagonism and it slightly improved, which might be due to the increase in the number of active bacteria. This cultivation system will have great reference significance for the future commercialization progress.

Ca^2+^ and Fe^3+^ have no obvious beneficial effect on the biofilm formation of JK-SH007. This is similar to the results of Wang (2014). Glycerol plays a decisive role in the ability of JK-SH007 to form a stable biofilm. The addition of glycerol to TSB medium produces a mature biofilm. When glycerin is added to LB medium, it is necessary to continue adding a certain amount of metal ions to produce a visible biofilm. This finding is similar to that of Gao T et al. who used LBGM medium (LB medium + 1% [vol/vol] glycerol + 100 μM MnSO_4_) to promote *B. subtilis* biofilm formation (Gao et al. 2015). Different media have different carbon–nitrogen ratios, which leads to different effects on biofilm synthesis abilities. Zhang Yan. described that changes in the carbon–nitrogen ratio affects the protein and polysaccharide contents of extracellular polymers in biofilms, thereby affecting the bacterial coating and the formation of biofilms (Zhang et al. 2015).

The optimal temperature for the static culture of JK-SH007 to form a biofilm is 25 °C. Generally, the optimum growth temperature of JK-SH007 is 30 °C (Ren et al. 2010). The optimal temperature for biofilm formation is 25 °C, which is the most suitable temperature for low-temperature stress. When the temperature continues to decrease, it is not conducive to the growth of bacteria, resulting in a reduction in the number of bacteria. When the threshold of bacterial abundance is not reached, the biofilm formed is not sufficiently dense. An excessively high and low pH prevents the normal growth of strain JK-SH007. Under normal growth conditions, pH has a great influence on biofilm-forming abilities. When the pH was 5, the amount of biofilm produced was optimal, which may be due to its own bacterial reproduction under this condition, and the number of bacterial reached the threshold; this is an acid stress environment. Pavioni et al. described that with the pH value, the change in the ionization capacity of certain functional groups of extracellular multimers secreted by bacteria also change, leading to changes in their ability to flocculate (Pavoni et al. 1972). The optimized biofilm formation system was TSB medium + 1% glycerol + 50 mM MgSO_4_. The bacterial suspension cultured at 180 r/min and 28 °C for 14 h (absorbance 600 nm ≈ 1.1) resulted in the optimal concentration of biofilm formation. Compared with the normal LB and TSB media, the new medium enables the survival of the bacteria for a longer period of time, which provides new technical support for the research progress of the fermentation process of the biocontrol bacteria JK-SH007.

*SuhB*, identified in chromosome 2 of *B. pyrrocinia* JK-SH007, is highly similar to the *E. coli* gene *suhB* Ec, and the primary amino acid sequence of *suhB* Ec is highly similar to that of the inositol monophosphatase (IMPase) mammalian protein (Matsuhisa et al. 1995). IMPase is an essential enzyme for the synthesis of phosphatidylinositol. Michell et al. found that phosphatidylinositol is used to prepare membrane phospholipids for archaea (Michell 2011). The results showed that the expression of the *suhB* gene was upregulated in biofilm bacteria compared with planktonic bacteria, and the expression level of the *suhB* gene increased to 3.847 in the 14 h phase of JK-SH007 growth, while the expression level was upregulated during the 6 day period of stable mature biofilm formation to reach 4.137. During the process of JK-SH007 biofilm formation, the *suhB* gene may be highly expressed, thereby resulting in the secretion of a large amount of phospholipids, which are used in biofilm extracellular matrix synthesis and participate in the signal molecular conduction of the quorum sensing two-component system (Munnik and Nielsen 2011; Shewan et al. 2011).

The *ropN* gene is required for the motility and biofilm formation of certain gram-negative bacteria, which control the synthesis of alginate, flagella, etc. (Totten et al. 1990; Hmama et al. 2004; Saldías et al. 2008; Yip et al. 2010). The results of this experiment showed that *ropN* in JK-SH007 bacteria cultured in a normal TSB culture was significantly upregulated to 3.159. Compared with the mobility at 14 h in the Control, the motility of JK-SH007 in the TMG was greatly enhanced to cope with the amount of bacteria. Logically, the nutrient content of the medium after the bacterial number reaches the K value is gradually lacking. Compared with the Control at 14 h, in the TMG with multiple exogenous stresses, the expression level of the *ropN* gene was downregulated to 0.66 after 6 days. This may be because JK-SH007 is a motile bacterium that forms a biofilm that requires a single cell to stop moving and produces a large amount of extracellular matrix. The planktonic bacteria begin to aggregate and adhere to the cell-to-medium junction (Watnick and Kolter 1999; Lemon et al. 2007). The downregulation of the *ropN* gene reduces the motility of JK-SH007 and contributes to the formation of a stable biofilm.

Poplar canker disease is a serious concern and occurs due to its weak growth. Biological control can achieve sustainable control of poplar canker disease. In particular, the use of JK-SH007 as a biocontrol agent not only promotes the growth of host plants but also enhances the disease resistance of poplars, and its biosafety has been proven. However, in actual production, due to the complexity of the environment, and the natural life history of JK-SH007, which is mostly a floating state, its biocontrol effect will decrease considerably with time, and biofilm formation directly addresses these problems. This will provide more theoretical technical guidance for use of JK-SH007 as a biocide.

## Declarations

I would like to declare on behalf of my co-authors that any figure or text taken from another paper is clearly indicated with the full source and permission of the authors of said source. Besides, our manuscript has not been published previously, and not under consideration for publication elsewhere, in whole or in part. All the authors listed have approved the present submitted version. And does not involve ethical issues.

## Data Availability

All the data and materials have been provided in main manuscript.
